# Transforaminal endoscopic lumbar discectomy with two-segment foraminoplasty for the treatment of very highly migrated lumbar disc herniation: a retrospective analysis

**DOI:** 10.1186/s12893-024-02379-2

**Published:** 2024-04-16

**Authors:** Yao Zhang, Jipeng Song, Wancheng Lin, Lixiang Ding

**Affiliations:** Department of Spinal Surgery, Beijing Shijitan Hospital, Capital Medical University, No. 10, Tieyi road, Yangfangdian, Haidian district, Beijing, 100038 People’s Republic of China

**Keywords:** Migrated lumbar disc herniation, Foraminoplasty, Pediculectomy, Transforaminal endoscopic lumbar discectomy

## Abstract

**Background:**

The surgical resection of very highly migrated lumbar disc herniation (VHM-LDH) is technically challenging owing to the absence of technical guidelines. Hence, in the present study, we introduced the transforaminal endoscopic lumbar discectomy (TELD) with two-segment foraminoplasty to manage VHM-LDH and evaluated its radiographic and midterm clinical outcomes.

**Materials and methods:**

The present study is a retrospective analysis of 33 consecutive patients with VHM-LDH who underwent TELD with two-segment foraminoplasty. The foraminoplasty was performed on two adjacent vertebrae on the basis of the migration direction of disc fragments to fully expose the disc fragments and completely decompress the impinged nerve root. The operation duration, blood loss, intra- and postoperative complications, and recurrences were recorded. Additionally, imageological observations were evaluated immediately after the procedure via magnetic resonance image and computerized tomography. Clinical outcomes were evaluated by calculating the visual analog scale (VAS) score and Oswestry Disability Index (ODI). The MacNab criterion was reviewed to assess the patients’ opinions on treatment satisfaction. The resection rate of bony structures were quantitatively evaluated on postoperative image. The segmental stability was radiologically evaluated at least a year after the surgery. Additionally, surgery-related and postoperative complications were evaluated.

**Results:**

The average age of the patients was 56.87 ± 7.77 years, with a mean follow-up of 20.95 ± 2.09 months. The pain was relieved in all patients immediately after the surgery. The VAS score and ODI decreased significantly at each postoperative follow-up compared with those observed before the surgery (*P* < 0.05). The mean operation duration, blood loss, and hospital stay were 56.17 ± 16.21 min, 10.57 ± 6.92 mL, and 3.12 ± 1.23 days, respectively. No residual disc fragments, iatrogenic pedicle fractures, and segmental instability were observed in the postoperative images. For both up- and down- migrated herniation in the upper lumbar region, the upper limit value of resection percentage for the cranial SAP, caudal SAP, and pedicle was 33%, 30%, and 34%, respectively; while those in the lower lumbar region was 42%, 36%, and 46%, respectively. At the last follow-up, the satisfaction rate of the patients regarding the surgery was 97%. Surgery-related complications including dural tear, nerve root injury, epidural hematoma, iatrogenic pedicle fractures, and segmental instability were not observed. One patient (3%) suffered from the recurrence of LDH 10 months after the initial surgery and underwent revision surgery.

**Conclusions:**

The TELD with two-segment foraminoplasty is safe and effective for VHM-LDH management. Proper patient selection and efficient endoscopic skills are required for applying this technique to obtain satisfactory outcomes.

## Background

Lumbar disc herniation (LDH) with a series of clinical manifestations occurs because of intervertebral disc tissue degeneration caused by various reasons, which results in a dural sac or nerve root compression in the posterior spinal canal [1–3]. Disc fragment migration refers to the protruded nucleus pulposus extending beyond the inferior or superior margin of the intervertebral disc in the downward or upward direction [4, 5]. Lee et al. [4] classified disc migration, in which the disc fragment migrated above zone 1 and below zone 4 was classified as very highly upward- and downward-migrated LDH [6].

The surgical resection of the very highly migrated LDH (VHM-LDH) is difficult because approaching the migrated fragments is challenging, and there is a high possibility of disc fragments residual. Lee et al. [4] reported that the endoscopic lumbar discectomy using the transforaminal approach is unsuitable for treating distant upward migration because exposing disc fragments to the operating field is difficult. In agreement with Lee et al.’s conclusion [4], Choi et al. [7] suggested that open surgery might be more effective than minimally invasive techniques in VHM-LDH management. However, advanced endoscopic instruments and techniques have enabled the application of endoscopic lumbar discectomy to manage high-grade, especially VHM-LDH [8, 9]. Recently, several techniques such as a modified single-level foraminoplasty technique [7, 10], a transpedicular technique [11], a trans-facet process and pedicle-complex technique [12], and a suprapedicular technique [13] were reported to successfully access the distant upward- or downward-migrated herniations. However, these existing techniques have several limitations regarding their practicability, indication ranges, and iatrogenic segmental instability. Hence, herein, we introduced the transforaminal endoscopic lumbar discectomy (TELD) with two-segment foraminoplasty and reviewed its clinical and radiographic outcomes. We hope this technique will serve as an effective and reliable alternative to other techniques for treating VHM-LDH.

## Materials and methods

### Patient recruitment

**Fig. 1:**
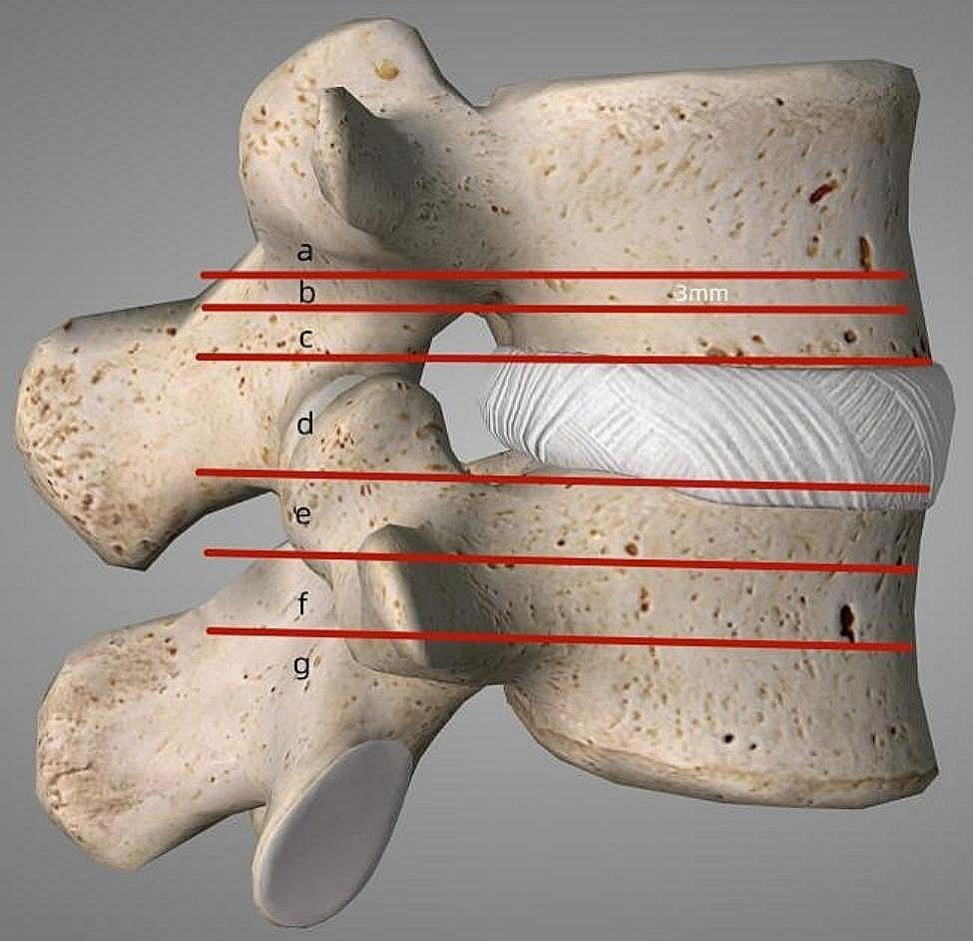
Illustration for the classification of migrated LDH by Lee et al. [4]; line a, b, and c indicates the boundary for upward very-highly, highly, and moderately migrated herniation, respectively (the distance between the line a and b is 3 mm; d indicates the intervertebral disc level; line e, f, and g indicates the boundary for moderately, highly, and very-highly downward herniation, respectively

The present retrospective study was approved by the ethics committee of the Beijing Shijitan Hospital of Capital Medical University. All patients provided written informed consent. Between January 2017 to December 2020, patients diagnosed with LDH and underwent TELD were reviewed. TELD indication was based on the pain referral pattern, compatible imaging results, positive physical signs, and poor response to previous standardized conservative treatment, such as physical therapy and oral medication. The evaluation of the extent of disc fragment migration was based on the study of Lee et al. [4] (Fig. 1). The inclusion criteria were as follows: (1) aged over 18 years, (2) had no spinal surgery history, (3) identified as VHM-LDH, (4) underwent the TELD with two-segment foraminoplasty, (5) had completed at least 1-year follow-up, (6) underwent magnetic resonance image (MRI) and computerized tomography (CT) for the lumbar spine immediately after the surgery, and (7) had flexion-extension radiography for the lumbar spine at least 1 year after the surgery. The exclusion criteria were as follows: (1) had incomplete follow-up data or imageological information, (2) had associated bony central or lateral recess stenosis, (3) had the calcified disc at the responsible level, (4) had segmental instability, (5) had migrated fragments behind the S1 vertebra with high iliac crest and thick transverse process, (6) had evidence of an infectious or neoplastic disease, and (7) underwent other surgical interventions as part of the treatment.

### Surgical technique

All surgeries were performed under local anesthesia with patients in prone positions on radiolucent tables using C-arm fluoroscopy. A total of 10 mL of 1% lidocaine was used for the infiltration anesthesia of the entry tract. Continuous feedback was obtained from the patients during the entire procedure to avoid neural structure damage.

### Preoperative evaluation and designation

**Fig. 2:**
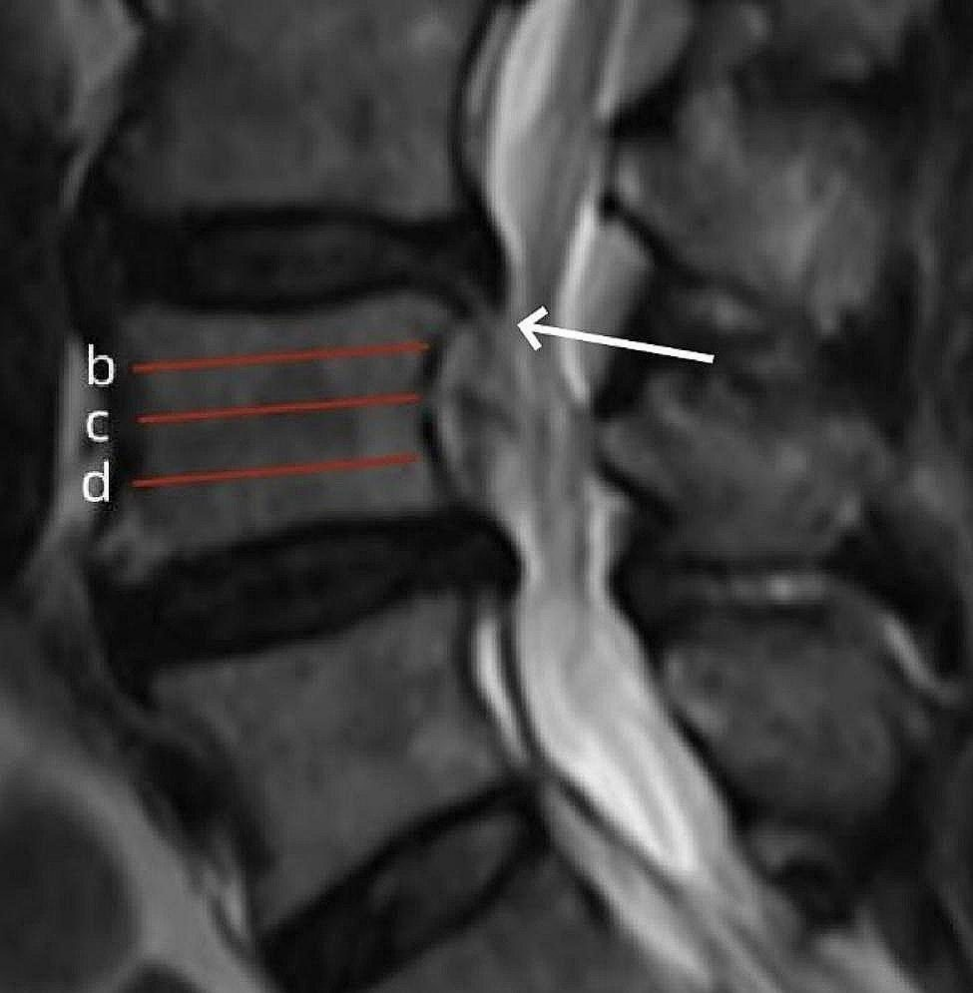
Preoperative evaluation on sagittal view of T2-weighted MRI. Line b, c, and d indicate the boundary for very-highly, highly, and moderately migrated herniation, respectively; the white arrow indicates the ruptured anulus fibrosus

The two-segment foraminoplasty and fragmentectomy were designed to be performed in the same longitudinal incision (1.5–2 cm) in order to minimize the collateral damage to soft and bony tissues. For both upward- and downward-migrated herniations, foraminoplasty at the responsible disc level should be performed initially. The responsible disc, where the fragments originate, was confirmed by finding the annulus fissure on T2-weighted MRI (Fig. 2). Axial MRI or CT was used to calculate the distance of the skin entry point of the needle from the posterior midline, and the needle trajectory aimed to target the migrated fragment while avoiding the posterior peritoneum.

### Downward-migrated herniations

In patients with very highly down-migrated herniation where the disc fragments migrate over the pedicle’s lower margin of the inferior vertebra, an appropriate pediculectomy is necessary to approach the fragments. Herein, for the first foraminoplasty on the responsible disc level, the puncture route of the needle on the lateral fluoroscopic view was set from the middle of the superior articular process (SAP) to the upper 1/3rd of the pedicle of the vertebra located where below the responsible disc, with a cranial to caudal inclination. After the staged dilation, the working cannula (8.4 mm) was docked to introduce an endoscope (6.3 mm) (Joimax, Karlsruhe, Germany).

The soft tissue around the peripheral facet joint was endoscopically ablated using a bipolar radiofrequency electrode (Guanlong, Shandong, China), and the SAP anatomic structure, pedicle, posterior vertebra, and intervertebral space were initially identified. Next, the ventral SAP was partially resected using an endoscopic trephine and Kerrison punch to enlarge the intervertebral foramen. We recommend performing step-by-step facetectomy to ensure that the endoscope reaches the anterior epidural space while avoiding excessive SAP resection. The dural sac, traversing nerve root, and partial disc fragments were visualized at this stage. The intradiscal decompression and debulking of the intervertebral disc were performed before the fragmentectomy to create enough anterior epidural space. A partial pediculectomy was performed where the upper medial border of that pedicle was resected to access the migrated disc fragments more appropriately. An endoscopic drill with a round diamond burr tip was used at this stage owing to its fine and delicate drilling capabilities. After these preparations, the end of the endoscope was cranially tilted to investigate the media pedicle and the superior half part of the vertebra, a retrievable part of the disc fragments. If the fragment was not sequestered, it was dragged and retrieved by holding its tail. If not, the axilla between the traversing nerve root and the dural sac was investigated; thus, the second foraminoplasty and fragmentectomy were necessary.

The second foraminoplasty and fragmentectomy were performed on the inferior foramen to investigate the axilla between the traversing nerve root and the dural sac and to retrieve the sequestered fragments hidden behind the inferior half part of the vertebra. After withdrawing the working cannula and endoscope, the needle was introduced to target the tip of the SAP of the inferior vertebra within the same incision. At this stage, foraminoplasty was needed to resect the tip or superior 1/3rd of the SAP. After the foraminoplasty, the end of the endoscope was tilted caudally for further investigation, and the sequestered fragments were observed lying in the axilla under the exiting root partly covered by the superior foraminal ligament and ligamentum flavum. After releasing these ligaments, the fragments were removed with forceps. The healthy intervertebral disc at this level was kept intact.

### Upward-migrated herniations

**Fig. 3:**
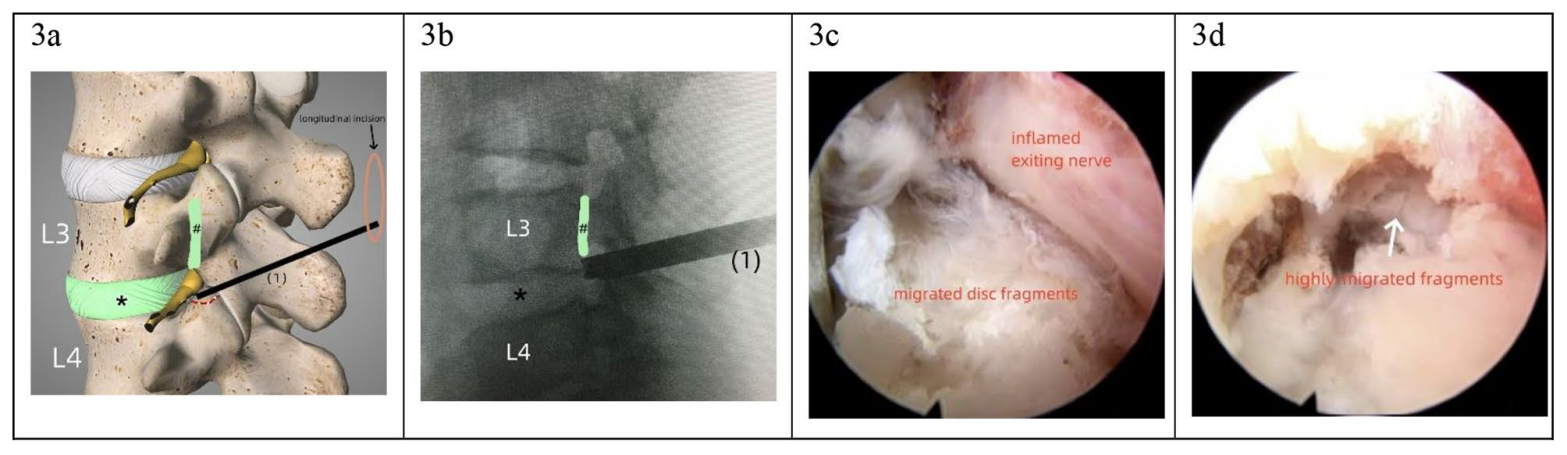
The first step foraminoplasty and fragmentectomy in very-highly upward-migrated herniation. *, the responsible disc level, #, the upward-migrated disc fragments. (**a**): Schematic representation of the first-step foraminoplasty for upward-migrated herniation at L3/4, the dotted red line indicates the margin of bony resection; (**b**): Lateral intraoperative radiography, the working cannula is cranially shifted for exploring the sequestered fragment; (**c**): Under endoscopic view, the migrated fragments hidden behind the inflamed exiting nerve root is explored; (**d**): After the initial fragmentectomy, very-highly upward-migrated fragments in the “hidden zone” is beyond the reach, a second fragmentectomy is needed

In such patients, the needle was targeted to the tip or superior 1/3rd of the SAP (Fig. 3a). As the working cannula was placed more cranially than that in downward-migrated herniation, the inflamed exiting root was carefully protected. The first foraminoplasty for upward-migrated herniations was performed by resecting the SAP tip, as the cannula was placed in the upper wider part of the foramen (Fig. 3b). Additionally, foraminoplasty was spared in a higher disc level with enough foramen space, such as L2/3 or L3/4. After the foraminoplasty, a round-end cannula was used to safely retract the exiting root and surrounding soft tissues. Intradiscal decompression was performed prior to the fragmentectomy to debulk the responsible disc and enlarge the anterior epidural space. After the initial investigation of the epidural space, the endoscope was gradually shifted upwards with a twisting gesture till the exiting root was partially visible. At this point, the sequestered fragments were observed that were hidden in the axilla between the exiting nerve and the dural sac (Fig. 3c). As the upward-migrated herniation was commonly sequestered, the fragments were retrieved in a piece-by-piece manner.

**Fig. 4:**
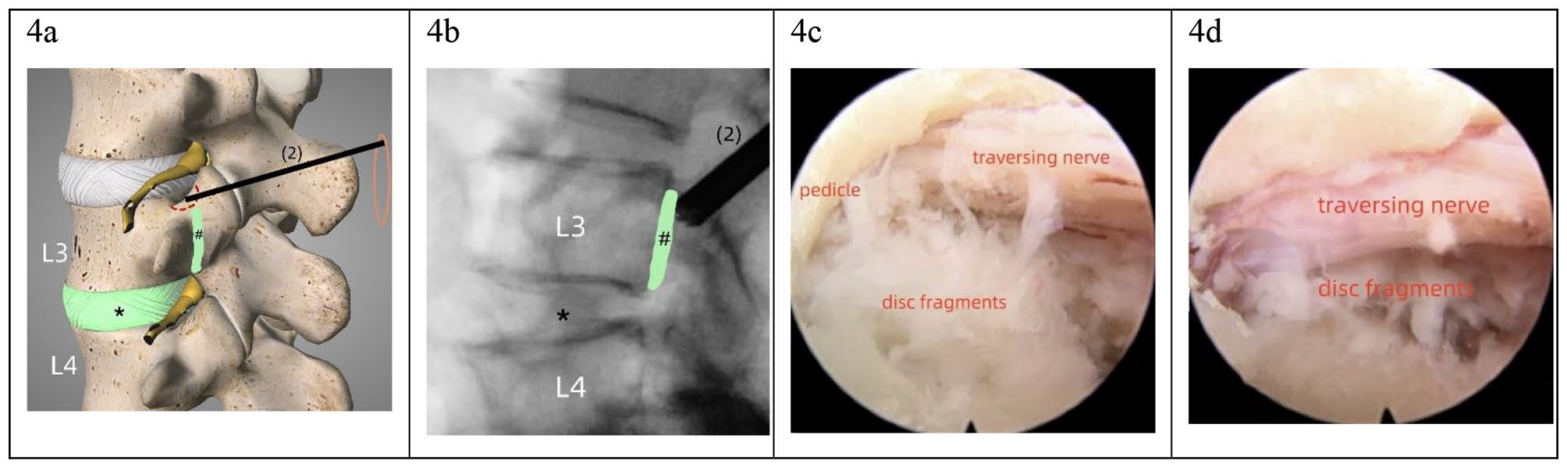
The second-step foraminoplasty and fragmentectomy in very-highly upward-migrated herniation. *, the responsible disc level, #, the upward-migrated disc fragments. (**a**): Schematic representation of the second-step foraminoplasty for upward-migrated herniation at L3/4, the dotted red line indicates the margin of bony resection; (**b**): An endoscopic trephine or round diamond burr was used during the second foraminoplasty, the endoscope was cranially inclined to explore the medial pedicle; (**c**), (**d**): After a partial pediculectomy and a caudal shift of the endoscope, the highly migrated fragments hidden in the medial pedicle were exposed and retrieved

The second foraminoplasty was usually necessary in very highly upward-migrated herniations because the fragments were often sequestered and the medial pedicle (the hidden zone) could not be approached (Fig. 3d). Foraminoplasty was performed on the cranial vertebra. The needle was advanced and located at the junction of the pedicle and the SAP base (Fig. 4a, b). Foraminoplasty and fragmentectomy procedures at this stage were similar to those in the downward-migrated herniations; however, bony destruction was less. The sequestered fragments hidden in the medial pedicle were identified and retrieved (Fig. 4c, d). Additionally, the healthy disc at this level was not infringed.

After careful investigation, pressure control by intermittently blocking the irrigation fluid outflow with the thumb allowed the traversing nerve root to move freely, which confirmed complete decompression.

### Technical key points

1. Identifying the origination of the disc fragments (the responsible disc) is sometimes difficult in very highly migrated herniations. In such cases, finding the annulus fissure on the sagittal view of a T2-weighted image is helpful.
2. For both migrated types, fragmentectomy at the responsible disc level should always be accomplished first.
3. Most of the disc fragments should be retrieved during the first-step fragmentectomy, and the second-step fragmentectomy is regarded as an auxiliary decompression.
4. Step-by-step bony resection is required for the two-segment foraminoplasty to minimize the damage to bony stabilizers.
5. Intradiscal decompression on the responsible disc is needed for acquiring enough epidural space, whereas the healthy disc should be kept intact.

### Clinical outcomes evaluations

The clinical outcomes were assessed before the surgery and at 1 week, 1 month, 6 months, 1 year, and 18–24 months (the last follow-up) after the surgery. The visual analog scale (VAS) score was calculated to assess the back and leg pain, and the Oswestry Disability Index (ODI) was calculated to assess the functional capacity. The modified MacNab criterion was followed to evaluate patients’ satisfaction regarding the surgery at each follow-up. Additionally, operating times, blood loss, intra- and postoperative complications, the hospital stays, the recurrences and the need for revision surgery were recorded.

### Imageological outcomes evaluations

**Fig. 5:**
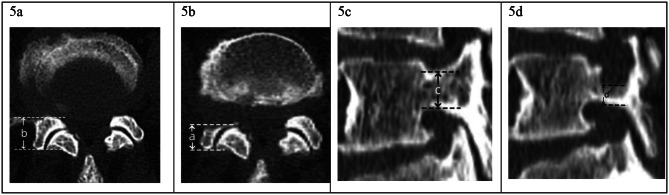
Calculation of the resection range rate of facet joint and pedicle. (**a**), (**b**): Resection range rate measured on axial CT image obtained before (left) and immediately after the surgery (right), the equation is (b-a)/b; (**c**), (**d**): Resection range rate measured on sagittal CT image obtained before (left) and immediately after the surgery (right), the equation is (c–d)/c

**Fig. 6:**
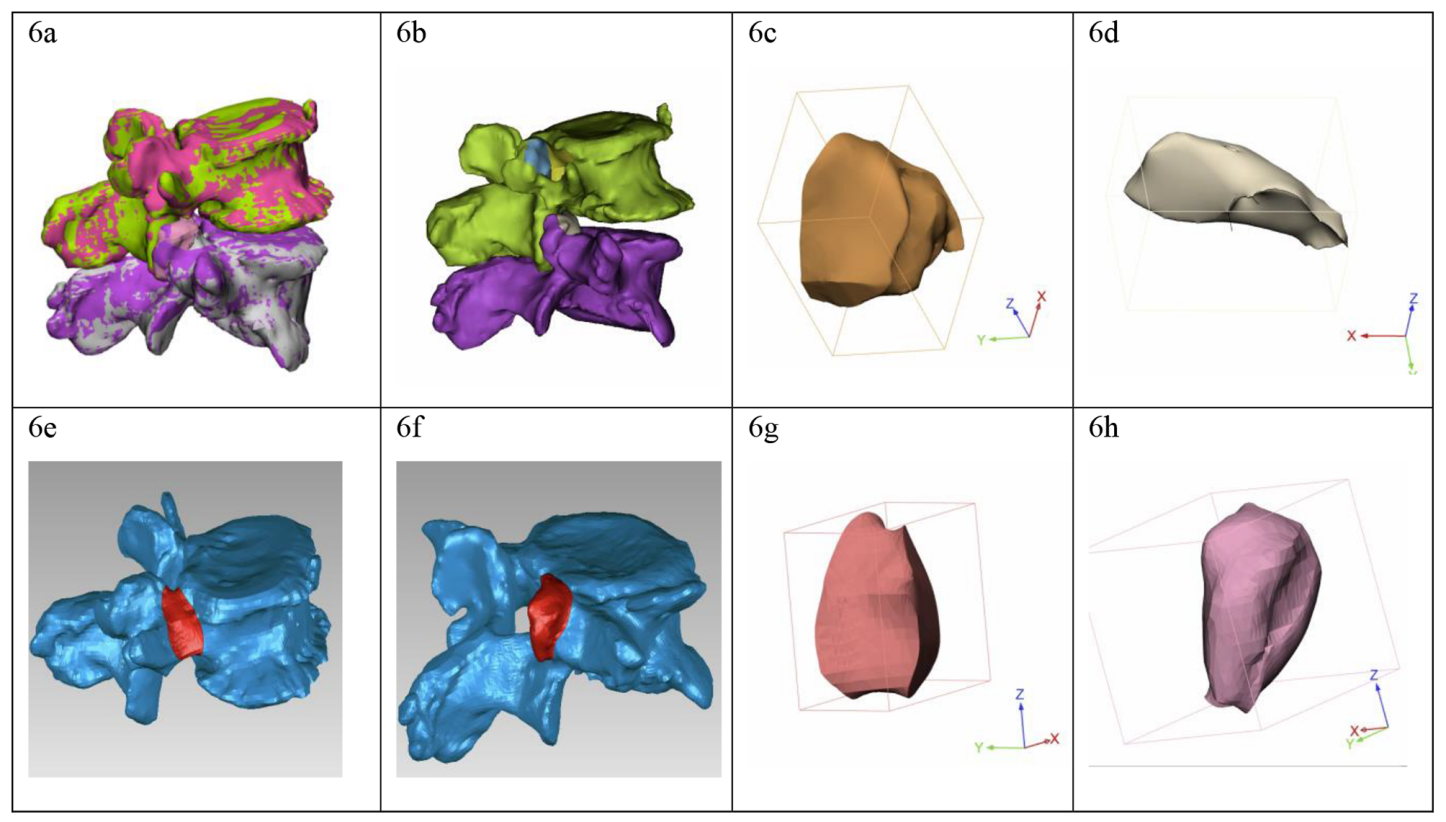
Calculation for the volume of pedicle and facet joint. (**a**): Image fusion of the pre- and post-operative 3-dimensional reconstructive vertebrae model; (**b**): The resection parts were obtained with Boolean operation; (**c**), (**d**): The model of resected pedicle (c) and SAP (d) were isolated, the volume was calculated; (**e**), (**f**): The preoperative pedicle (c) and SAP (d) were marked and obtained on Geomagic software; (**g**), (**h**): The model of the preoperative pedicle (g) and SAP (h) were obtained, the volume was calculated

Immediate postoperative CT and MRI images were captured for each patient to quantitatively evaluate the resection range and volume of the facet joints and pedicle, as well as the completeness of decompression. As illustrated in Fig. 5a and b, the resection range rate of facet joint from the cranial and caudal vertebral segment was calculated on the axial CT image, using the following equation: (length of preoperative facet-length of postoperative facet)/length of preoperative facet. The resection range rate of pedicle was calculated on the sagittal CT image by using the following equation: (length of preoperative pedicle–length of postoperative pedicle)/length of preoperative pedicle, as shown in Fig. 5c and d. The resection volume rate of facet joints and pedicle were calculated on Materialise’s interactive medical image control system (Mimics, version 21.0, Materialise, Switzerland) and Geomagic software (version 14, North Carolina, America). The Digital Imaging and Communications in Medicine (DICOM) data of pre- and post-operative CT image were input into Mimics and Geomagic software, the 3-dimensional reconstruction models of the operated vertebrae were obtained by using the “Calculate part” order on the selected mask; the pre- and post-operative vertebrae models were merged by using the “Global Registration” order (Fig. 6a), the resected part of pedicle and SAPs were obtained by using Boolean operation (Fig. 6b). The resected pedicle and SAP from the cranial vertebral segment were separated by using “Trim” order, the volume of theses parts were calculated (Fig. 6c, d). Then, the models of the preoperative pedicle and two SAPs were isolated on Geomagic software (Fig. 6e, f), the volume of each part was obtained (Fig. 6g, h). The resection volume rate of either facet joint or pedicle was calculated by using the following equation: volume of resected bone/volume of intact bone. An X-ray of the lumbar spine was taken at least 1 year after the surgery to evaluate segmental stability, in which an anterior translation of more than 3 mm between the vertebra where superior and inferior to the responsible disc level was considered radiological instability.

### Statistics

Collected quantitative variables were calculated and represented as means ± standard deviations (x ± sd), whereas obtained qualitative variables were calculated and represented as numbers and percentages. The VAS score and ODI measured before and after the surgery and at each follow-up were compared using repeated-measures analysis of variance. Values at *P* < 0.05 were considered statistically significant. All analyses were performed using SPSS 26.0 (SPSS, Inc., Chicago, IL, USA).

## Results

In total, 33 patients (23 males), with a mean age of 56.87 years (range: 44–77 years) fulfilled the inclusion criteria and were retrospectively analyzed. The mean follow-up period was 20.95 ± 2.09 months (range: 18–24 months). The L4 vertebra was the commonest segment where the disc fragment was located (16 patients, 48.4%). Furthermore, more downward-migrated disc fragments were found (20 patients, 60.6%) than upward-migrated (13 patients, 39.4%). The demographics and baseline characteristics are presented in Table 1.

**Table 1:** The demographic and baseline characteristics of all patients (*N* = 33)

Variables	Outcomes
**Age (mean ± SD) (years)**	56.87 ± 7.77
**Gender (n)**	
Male	23
Female	10
**Body Mass Index (mean ± SD)**	24.89 ± 3.01
**Chronic comorbidity (n)**	
Hypertension	7
Diabetes mellitus	4
**Direction of fragments migration (n) (percentage)**	
Upward	13 (39.4%)
Downward	20 (60.6%)
**Location of fragments (n) (percentage)**	
L2 vertebra	2 (6.1%)
L3 vertebra	14 (42.4%)
L4 vertebra	16 (48.5%)
L5 vertebra	1 (3.0%)
**Total**	33 (100%)

SD: standard deviation

The mean VAS score of leg and back pain significantly decreased at the 1-week postoperative follow-up period compared with that at the preoperative follow-up period (7.68 ± 0.88 vs. 2.11 ± 0.97, 3.76 ± 1.40 vs. 1.87 ± 0.95, *P* < 0.0001). Additionally, during the 1-year postoperative period and the last follow-up period, the VAS score of leg and back pain significantly decreased compared with those at the 1-week postoperative follow-up period (*P* < 0.0001). The same variation was found in the evaluation of the ODI score (Table 2). Based on the MacNab criteria, satisfactory (excellent or good) results were found in 88% of patients at the 1-week postoperative follow-up and 97% at the last follow-up period (Table 3).

**Table 2:** Comparison of VAS and ODI scores at each follow-up and pre-operation (*N* = 33)

	Preoperative	1-week	3-month	6-months	1-year	The last follow-up	P^1^	P^2^	P^3^
VAS^1^	7.68 ± 0.88	2.11 ± 0.97	1.34 ± 0.51	0.89 ± 0.24	0.65 ± 1.57	0.51 ± 0.23	*P* < 0.0001	*P* < 0.0001	*P* < 0.0001
VAS^2^	3.76 ± 1.40	1.87 ± 0.95	0.96 ± 0.63	0.43 ± 0.28	0.55 ± 0.92	0.23 ± 0.10	*P* < 0.0001	*P* < 0.0001	*P* < 0.0001
ODI	59.25 ± 10.82	35.85 ± 6.14	25.62 ± 7.04	14.62 ± 7.65	13.92 ± 5.36	12.29 ± 3.96	*P* < 0.0001	*P* < 0.0001	*P* < 0.0001

Pre-op: preoperation; VAS^1^ indicates the leg pain score, VAS^2^ indicates the lower back pain score; *P*^1^: comparison between the values at preoperative follow-up and the values at the 1-week follow-up; *P*^2^: comparison between the values at the 1-week follow-up and the values at the 1-year follow-up; *P*^3^: comparison between the values at the 1-week follow-up and the values at the last follow-up

**Table 3:** The patient’s opinion on treatment satisfaction (*N* = 33)

	Excellent	Good	Fair	Poor	Total
1-week	11 (0.33)	18 (0.55)	4 (0.12)	0 (0.00)	33 (1.00)
3-month	16 (0.48)	15 (0.45)	2 (0.07)	0 (0.00)	33 (1.00)
6-month	20 (0.61)	13 (0.39)	0 (0.00)	0 (0.00)	33 (1.00)
12-month	25 (0.76)	7 (0.21)	1 (0.03)	0 (0.00)	33 (1.00)
The last follow-up	23 (0.70)	9 (0.27)	1 (0.03)	0 (0.00)	33 (1.00)

**Fig. 7:**
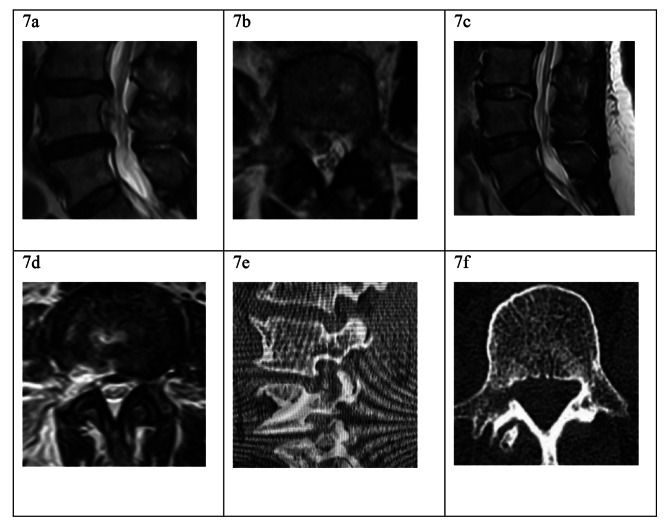
A case presentation for very-highly downward-migrated herniation. (**a**): The preoperative sagittal MRI showing a very-highly down-migrated fragment along the posterior L4 vertebra; (**b**): The preoperative axial MRI showing the migrated fragments hidden in the L4 medial pedicle; (**c**), (**d**): The postoperative sagittal and axial MRI showing that the fragments were completely retrieved, the L4/5 disc was maintained intact; (**e**), (**f**): The postoperative sagittal and axial CT showing the partial pediculectomy, while there is no pedicle fracture

All patients showed complete retrieval of the migrated disc fragments based on the postoperative MRI results, no patient showed iatrogenic pedicle or facet joint fracture (Fig. 7). With regard to the quantitative evaluation of bony resection, the up-migrated herniation at L2 vertebra required the smallest bony resection: the mean resection range rate of cranial SAP, caudal SAP, and pedicle was 17%, 20%, and 19%, respectively; while the mean resection volume rate of cranial SAP, caudal SAP, and pedicle was 20%, 24%, 17%, respectively. The down-migrated herniation at L5 vertebra has the largest bony resection, in which the mean resection range rate of cranial SAP, caudal SAP, and pedicle was 42%, 36%, and 45%, respectively; while the mean resection volume rate of cranial SAP, caudal SAP, and pedicle was 41%, 35%, 46%, respectively. An increasing tendency for the bony resection range and volume rate of bony structures were observed from L2 to L5 in both up- and down-migrated herniation. Moreover, at each vertebral level, when comparing to the down-migrated type, the up-migrated herniation had a larger resection range and volume rate of the caudal SAP, and a smaller resection range and volume rate of both the cranial SAP and pedicle (Table 4). The largest resection percentage for each bony structure is summarized in Table 5.

**Table 4:** Quantitative evaluation of bony resection range rate and volume rate (*N* = 33) (mean ± standard deviation)

	L2		L3		L4		L5	
	Up (*n* = 1)	Down (*n* = 1)	Up (*n* = 4)	Down (*n* = 9)	Up (*n* = 8)	Down (*n* = 9)	Up (*n* = 0)	Down (*n* = 1)
Resection range rate of facet joint^a^	0.17	0.22	0.21 ± 0.04	0.31 ± 0.06	0.29 ± 0.10	0.36 ± 0.11	NA	0.42
Resection range rate of facet joint^b^	0.20	0.18	0.28 ± 0.05	0.26 ± 0.05	0.32 ± 0.09	0.28 ± 0.05	NA	0.36
Resection range rate of pedicle	0.19	0.25	0.22 ± 0.05	0.29 ± 0.08	0.34 ± 0.03	0.40 ± 0.07	NA	0.45
Resection volume rate of facet joint^a^	0.20	0.23	0.23 ± 0.06	0.33 ± 0.09	0.31 ± 0.08	0.38 ± 0.09	NA	0.41
Resection volume rate of facet joint^b^	0.24	0.21	0.30 ± 0.09	0.28 ± 0.07	0.35 ± 0.08	0.32 ± 0.05	NA	0.35
Resection volume rate of pedicle	0.17	0.27	0.29 ± 0.10	0.34 ± 0.11	0.31 ± 0.12	0.43 ± 0.08	NA	0.46

Up: up-migrated herniation; Down: down-migrated herniation; NA: not applicable

^a^The facet joint of the cranial vertebral segment

^b^The facet joint of the caudal vertebral segment

**Table 5:** The largest resection percentage for each bony structure at each vertebral segment (*N* = 33)

	L2		L3		L4		L5	
	Up (*n* = 1)	Down (*n* = 1)	Up (*n* = 4)	Down (*n* = 9)	Up (*n* = 8)	Down (*n* = 9)	Up (*n* = 0)	Down (*n* = 1)
Facet joint^a^ (mean) (percentage)	20%	23%	23%	33%	31%	38%	NA	42%
Facet joint^b^ (mean) (percentage)	24%	21%	30%	28%	35%	32%	NA	36%
Pedicle (mean) (percentage)	19%	27%	29%	34%	34%	43%	NA	46%

Up: up-migrated herniation; Down: down-migrated herniation; NA: not applicable

^a^The facet joint of the cranial vertebral segment

^b^The facet joint of the caudal vertebral segment

All surgeries were performed successfully. The mean operation duration and blood loss were 56.17 ± 16.21 min (range: 43–74 min) and 10.57 ± 6.92 mL, respectively. The mean hospital stay was 3.12 ± 1.23 days (range: 2–5 days). No surgery-related complication, including dural tear, nerve root injury, epidural hematoma, wound infection, or segmental instability, was found during the follow-up. Two patients experienced early postoperative dysesthesia of the traversing root that was satisfactorily treated using the neurotrophic drug for 2 weeks. One patient (3%) experienced the recurrence of lumbar disc herniation 10 months after the initial surgery and recovered completely after undergoing revision surgery with posterior short-segment fixation and interbody fusion.

## Discussion

Disc fragment migration can be frequently observed in clinical practice, accounting for 35–72% of cases [14, 15]. Although some researchers report that cranial and caudal migration occurs at the same frequency. some researchers have shown that more herniation is displaced downward than upward [16], which is consistent with our findings (downward migration of 60.6%). The risk of disc fragment residual during surgery in the cranial-migrated LDH is theoretically high because up-migrated herniations are prevalently sequestrated and multiply fragmented [16]. Contrarily, far-migrated disc fragments sequester under the cranial lamina and medial to the pedicle, which is considered a hidden zone LDH [6, 7, 17].

Traditionally, the open interlaminar is considered a standard approach to surgically managing LDH. Nevertheless, in the highly migrated LDH, an interlaminar approach requires hemilaminotomy or hemilaminectomy of the cranial or caudal vertebra, which is usually associated with partial or complete resection of facet joints, resulting in an increased risk of segmental instability [18, 19]. To minimize the possibility of iatrogenic segmental instability, Di Lorenzo et al. [20] used a microscopic translaminar technique. In this technique, a drill hole was made on the lamina just above the sequestration. Papavero et al. [21] described this technique as “hooking fish”. Nevertheless, due to the limitation of the surgical view, it is difficult to view the far-migrated disc fragments and the intervertebral space using the translaminar technique. Wang et al. [17] could access the migrated disc fragment by partially resecting the lateral lamina, which was named as an extra-laminar approach. This technique can avoid the destruction of facet joints; however, the intervertebral discectomy for the downward-migrated LDH is not feasible.

The percutaneous endoscopic lumbar discectomy technique (PELD) has immensely advanced in the last decade [8]. Some previous contraindications have now become surgical indications [22–24]. Many endoscopic techniques have been developed to treat far-migrated LDH. Lee et al. [4] reported the use of the “epiduroscopic” technique (outside-in) after foraminoplasty to manage high-grade upward and downward-migrated LDH. All procedures should begin from the disc space with an annular cutting procedure to properly access the fragments and avoid the violation of neural elements due to the direct approach to the epidural space. Nevertheless, they did not describe in detail the skills required for performing foraminoplasty in downward-migrated type LDH.

Choi et al. [1] reported the pediculectomy using an endoscopic reamer or a drill to perform foraminoplasty in highly downward-migrated LDH. They report that the annulus should not be penetrated via the cannula during the complete surgical procedure to prevent damage to the central disc. The method described by Choi et al. is different from that described by Lee et al. The limitation of this technique was the difficulty in managing the downward-migrated sequestrations that invade the axilla between the traversing nerve root and the dural sac. In their study, no patient suffered from neurologic injury or cerebrospinal fluid leak, and 2 of 59 patients (3.4%) underwent revision surgery. Cai et al. [25] reported a case series of 37 patients with high-grade downward-migrated LDH who were treated using an endoscopic foraminoplasty technique. In their study, one patient suffered recurrent radicular pain at the same segment three days postoperatively, and a second TELD was performed for revision. Yang et al. [26] treated 41 patients with far-downward-migrated discs using TELD. In this technique, a bone drill was used under fluoroscopic monitoring to complete foraminoplasty. They reported that three patients (7.3%) suffered a dural tear and one patient (2.4%) suffered a recurrence.

Apart from the standard transforaminal approach, a few researchers have reported many novel alternatives. Hu et al. [12] performed an osteotomy involving the facet process of SAP and the pedicle complex with trephine to enlarge the foramen for accessing the highly downward-migrated fragment. This foraminoplasty procedure was monitored under a C-arm fluoroscope rather than an endoscopic view; therefore, the potential risk of nerve root injury was high. Additionally, they did not report the application of this technique in patients with upward-migrated LDH. Xia et al. [27] reported the use of PELD via a posterior vertical anchoring technique to treat 13 patients with highly upward-migrated discs. In their study, a 9-mm bony hole was constructed in the middle part and inferomedial margin of the cranial lamina via an endoscopic drill. They reported a 92.3% satisfactory outcome. Xia et al.’s technique was based on the microscopic translaminar approach previously reported by Schulz et al. [28] and Soldner et al. [29]. However, in comparison of the microscopic technique, the endoscopic translaminar approach is able to provide a clearer surgical view, enabling a sufficient retrieval of disc fragments that both highly up- and down-migrated [27,068,038]. Among these novel approaches, the endoscopic translaminar technique is the most minimally invasive one, without any disturbances to facet joint and pedicle. Nonetheless, the bony hole should be properly prepared to avoid the iatrogenic pars fracture, which is much technically demanding; besides, it is incapable when the intervertebral space should be managed.

Kim et al. [30] reported the transforaminal PELD technique via a contralateral approach. Using this technique, the extremely down-migrated fragments can be removed without much cephalic-caudal endoscope trajectory. Furthermore, the contralateral transforaminal technique possessed several advantages. The conventional ipsilateral TELD is difficult to perform due to anatomical limitations, such as ipsilateral foraminal stenosis and low position of the exiting nerve root. Liu et al. [31] compared the clinical outcomes of the endoscopic interlaminar, transforaminal, and contralateral transforaminal approaches for the treatment of highly migrated discs, both upward and downward. They reported that the contralateral transforaminal approach has the longest operative time (mean: 112 min) and fluoroscopy time (mean: 14.6 min). Furthermore, one patient suffered iatrogenic cauda equina injury. The contralateral transforaminal approach allows the removal of the cranially migrated disc fragment, avoiding the osseous barrier by the pedicle, but this approach is difficult to perform. Furthermore, it is dangerous to approach the fragments via the anterior epidural space from the contralateral foramen.

Determining the responsible disc level is essential for determining the order of decompression and planning the skin incision when performing our technique. The fragmentectomy at the responsible disc level should be considered as the key procedure and should be completed in the first place, which is crucial for retrieving the main part of the migrated disc fragments. Subsequently, the secondary fragmentectomy at the adjacent disc level can allow a complete removal of any remaining fragments hidden in the “hidden zone” and the axilla between the traversing nerve root and the dural sac. It should be noted that the second step of fragmentectomy in our technique should be regarded as a kind of supplement procedure, as under certain circumstance where a highly up-migrated disc fragment is not fully sequestered, a single-segment TELD is sufficient to complete the decompression. Throughout this study, the extent of fragment removal was validated through postoperative imaging assessments in all patients, which revealing no residual fragments. The long-term satisfaction rate reached 97%, which is comparable with open-microdiscectomy and other endoscopic techniques [4, 5, 7, 20, 21, 24]. One patient (3%) experienced herniation recurrence and underwent revision surgery during the 10-month follow-up. The revision rate was low, and the time interval between the two operations was acceptable.

While our technique enables clear exploration of the impinged nerve root and ensures reliable decompression, it is important to acknowledge that it is technically demanding, moreover, this technique requires more bony resection than others existing approaches that used for retrieving highly migrated disc. Accordingly, we quantitatively analyzed the bony resection, including the resection range and the volume. On the basis of the present study, we observed an increasing tendency for the resection of bony structures from L2 to L5 in both up- and down-migrated herniation, mainly due to the thickening of both pedicle and facet joints in the lower lumbar region. For both up- and down- migrated herniation in the upper lumbar region (L2 and L3), the upper limit value of resection percentage for the cranial SAP, caudal SAP, and pedicle was 33%, 30%, and 34%, respectively; while those in the lower lumbar region (L4 and L5) was 42%, 36%, and 46%, respectively. The removal of facet joints and pedicle were observed less than 50% in each vertebral segment, no instances of pedicle or facet joint fractures or segmental instability were observed during the available follow-up period. However, a quantitative bony resection can be challenging under an endoscopic view when comparing to an open approach; thus, a step-by-step bony undercutting technique for repeatable facetectomy or pediculectomy would be more practical. Although the step-by-step bony resection during foraminoplasty may be time-consuming, it can streamline the subsequent procedures and avoid the over disturbances to these bony stabilizers. In this case series, the average operation duration was 56.17 ± 16.21 min, ranging from 43 to 74 min, which is comparable to traditional TELD procedures [3, 4, 26]. No complication associated with the approach was observed during the follow-up period, including dural tear, nerve root injury, epidural hematoma, wound infection.

Our technique may expand the indications for TELD for treating far-migrated LDH, allowing the retrieval of both highly upward- and downward-migrated disc fragments. However, it is important to note that the retrieval of highly migrated herniations at the L5 and S1 levels is challenging compared with other levels. The presence of a high iliac crest, a thick transverse process, and periforaminal osteophytes can inhibit the entry of the working cannula. In such cases, an endoscopic interlaminar approach can be a simpler and more effective alternative.

The present study has some limitations that should be addressed. The non-randomized design and the absence of matching with a control or cohort group make it difficult to obtain definitive conclusions on the efficacy and advantages of the technique. The appropriate resection of the bony structures during the two-segment foraminoplasty can streamline the next procedure. However, this method is technically demanding. Inadvertent over-resection of pedicle or facet joints can lead to segmental instability and potential neurological injury. Although we performed the quantitative evaluations for bony resection, a few cases with short-term follow-up can reduce the credibility of our present findings. Therefore, a large number of cases with prolonged follow-up period are warranted to investigate the long-term efficacy of our technique.

## Conclusion

In this retrospective study, our findings reveal that TELD with two-segment foraminoplasty is a safe and feasible surgical approach for managing the very highly upward- or downward-migrated LDH. This technique allows for the comprehensive decompression of the impinged nerve root throughout its course and allows the exploration and retrieval of hidden disc fragments. This technique can be considered as a supplement to single-segment TELD; however, it is important to note that it is technically demanding. A limited bony resection, with less than 50% of dagamge to pedicle and SAP, is important to avoid iatrogenic segmental instability. Further study aimed to investigate the learning curve and design a better quantitative foraminoplasty is needed.

## Data Availability

The data analyzed during the current study are available from the corresponding author upon a reasonable request.
